# Surveying the Efficacy of an Open Access Biomedical Informatics Boot Camp

**DOI:** 10.1055/a-2547-5208

**Published:** 2025-06-25

**Authors:** Skyler D. Resendez, Gillian Franklin, Crystal Tomlin, Rachel Stephens, Heather Maness, Srikar Chamala, Ross Koppel, Peter L. Elkin

**Affiliations:** 1 Department of Biomedical Informatics, University at Buffalo, Buffalo, New York, United States; 2 Center for Instructional Technology and Training, University of Florida, Gainesville, Florida, United States; 3 Department of Pathology and Laboratory Medicine, Children's Hospital Los Angeles, Los Angeles, California, United States; 4 Department of Pathology, University of Southern California, Los Angeles, California, United States; 5 Department of Biomedical Informatics, University of Pennsylvania, Philadelphia, Pennsylvania, United States; 6 Department of Veterans Affairs, Knowledge Based Systems and WNY VA, Buffalo, New York, United States

**Keywords:** education, clinical informatics, consumer health informatics, professional training, informatics specialists

## Abstract

**Objective:**

This study aimed to assess the efficacy of a biomedical informatics boot camp with regard to improving the skill sets of its participants.

**Methods:**

The University at Buffalo hosts a free, virtual biomedical informatics boot camp annually. Lectures covering various subject matters are offered, for example, general programming, machine learning, natural language processing, and clinical decision support. Once the 2023 boot camp had concluded, an anonymous voluntary survey was distributed.

**Results:**

Seventy percent of the survey respondents indicated that they agreed that their expectations were met. Eighty-two percent of the respondents indicated that our JupyterHub and the associated educational coding materials are useful tools for learning. Free response answers showed a desire for additional hands-on courses over theoretical lectures.

**Conclusion:**

The results were overwhelmingly positive. Most respondents felt they had expanded upon their knowledge of informatics. The study also pointed out challenges, including keeping difficulty levels appropriate for an audience with diverse educational backgrounds.

## Background and Significance

Educating a new generation of scientists in the field of bioinformatics and biomedical informatics poses a series of grand challenges. 1 By nature, research in these fields requires a highly interdisciplinary approach to achieve results as it incorporates various subjects, including, but not limited to, biology, medicine, computer sciences, and mathematics. Numerous studies have been conducted, illuminating these challenges as countries worldwide have attempted to incorporate informatics into their biological curriculums. 1 2 3 4 5 6 In the United States, lack of faculty expertise and training, lack of student interest, overly full curricula, and lack of student preparation have all created barriers to incorporating bioinformatics into life sciences education. 2 In this study, the researchers seek to evaluate the effectiveness of a biomedical informatics boot camp designed to alleviate some of these barriers.

Boot camps, being defined as short, intensive, and rigorous courses of training, are well-known for their effective strategy of improving educational outcomes for participants. 7 Participants across many specialties, including health care, have benefited from boot camps at different stages of their careers. 7 8 9 10 Overall, boot camps provide crucial information and practice to grant necessary skills, knowledge, and confidence. 7

Although boot camps are short and intensive, participants can acquire new skill sets. 11 New skill sets can be acquired across the continuum of biomedical informatics. These range from developing data analytic skills that can be used as biomedical informatics tools to the ability to undertake research in biomedical informatics, 12 both of which can address various challenges in the field.

The rapidly expanding field of biomedical informatics requires professionals to be prepared to meet the growing demands. 13 Knowledge and skills gained from continuous improvement prepare trainees and professionals alike to share a role in the continuum of change by relying on learned scientific principles and evidence that leads to improved outcomes. 14 Furthermore, scientists must evaluate all educational methods to meet the ever-evolving demands of teaching biomedical informatics.

## Objectives

The goal of this study was to assess and critically review the efficacy of a free, virtual biomedical informatics boot camp with regard to its ability to improve the skill sets of its participants.

## Methods

### Intervention

Each summer, the University at Buffalo hosts a free, virtual biomedical informatics boot camp. Anyone interested in biomedical informatics is allowed to attend. This study seeks to evaluate the effectiveness of the 2023 boot camp after offering lectures covering various subject matters, including, but not limited to, machine learning, natural language processing, cybersecurity, programming, database queries, clinical decision support, statistics, ontology, and public and consumer health informatics.

The boot camp consisted of 23 courses held between July 5 and August 21, 2023. The program was led by 13 instructors, each of whom volunteered to teach between one and four courses. These instructors have all been trained in the field of biomedical informatics with varying areas of expertise. Furthermore, these instructors were carefully selected to offer a wide array of topics of study.

Each course was scheduled for 1.5 hours, totaling approximately 34.5 hours. The lectures were also recorded and uploaded to allow for asynchronous viewing. Metrics were collected concerning the number of participants that attended each lecture and the number of unique participant usernames that accessed each recording.

Most of the materials used during the boot camp are stored in a JupyterHub that is available to all who request access. This JupyterHub contains a rich repository of information and codes designed to help teach biomedical informatics. While some of the codes stored here are used throughout the boot camp, there are additional codes, tutorials, data files, and lectures that serve as supplementary materials. These materials cover topics such as python and R coding, SPARQL Protocol and Resource Description Framework Query Language (SPARQL), general statistics including linear regression, natural language processing, and machine learning.

### Survey Methodology

At the conclusion of the boot camp, an anonymous, voluntary survey was offered to everyone who signed up for the program. The survey was created and administered online. Emails were sent to all who signed up for the boot camp with instructions on where to find the survey and how to fill it out. The email informed everyone that the survey was entirely anonymous, voluntary, and designed for both academic study and quality improvement (QI) purposes. To ensure consent, the sole required question prior to the survey screen was “Do you consent to the answers you provide here being used in an academic study? Your participation in this survey is completely voluntary and your responses are anonymous. You have the right to refuse to take part in this survey or choose to stop without judgment or penalty. You can choose to decline answering any particular question that you do not wish to answer for any reason.” The responses were either “Yes, I do” or “No, I do not,” with the latter resulting in the survey ending before any additional questions were displayed.

The survey consisted of 11 questions, 3 of which had two subsections. The survey contained both selected response and free response questions. Question topics included the effectiveness of the boot camp and the materials used during the duration of the boot camp, suggestions for improvement, and the responsiveness of our instructors. Supplementary Table S1 (available in the online version only) shows the questions contained in the survey.

Counts and percentages (based on the total number of responses) were calculated for selected response results. Trends in free response answers were initially observed using Microsoft Copilot. These trends were used to create categories for different types of responses, and counts for each category were tallied by reading through each answer manually.

## Results

Over 400 individuals signed up for the 2023 University at Buffalo biomedical informatics boot camp, including 46 U.S. institutions, National Institutes of Health (NIH) personnel who attended the boot camp, and participants from other countries.

An average of just over 38 participants attended each lecture (high of 124). Further, an average of just over 50 participants accessed each recording of the lectures (high of 118). The metrics for these courses can be found in Table 1 .

**Table 1:** Participant counts for each boot camp course

Class name	Live lecture participants	Unique recording views
Introduction to Python Programming	124	118
Machine Learning—Logistic Regression and Neural Networks	72	106
Machine Learning—Decision Trees and Random Forest	57	81
Natural Language Processing	66	77
R-Studio Basics and Regression Analysis	53	55
Structural Bioinformatics and Drug Discovery	35	59
Translational bioinformatics—Computational analysis of Novel Drug Opportunities	22	59
Medical Terminology and Standards	39	41
Human Factors Engineering	50	40
Elements of Logic for Ontology Design	31	45
Cybersecurity and Qualitative Research Methods	37	38
Realism-based Biomedical Ontology	21	37
Biomedical Ontology—SPARQL Queries	33	44
Structured Query Language	23	36
Introduction to Unix/Linux Programming	28	32
Introduction to Precision Genome Informatics	19	35
The place of Referent Tracking in Biomedical Informatics	26	28
Use of artificial intelligence in medical teaching, medical care, and medical research	24	43
Public Health Informatics	23	37
Modeling and HL7 (including Fast Healthcare Interoperability Resources)	25	38
Consumer Health Informatics	28	30
Image Analytics	20	30
Clinical Decision Support	23	49
Average	38.22	50.35

Abbreviation: SPARQL, SPARQL Protocol and Resource Description Framework Query Language.

Each participant who signed up for the boot camp was invited to participate in the survey. We received at least partial responses from 68 adult respondents. These were used in our analysis. Of these respondents, 22 indicated that they were clinical informatics fellows, and 21 indicated that they were doctoral-level students.

**Fig. 1:**
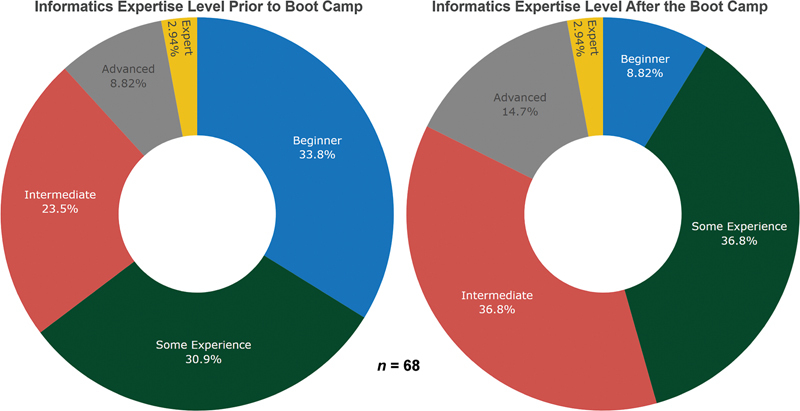
Respondent self-analysis of informatics expertise level before and after the 2023 University at Buffalo Biomedical Informatics Boot Camp.

The results of the boot camp were overwhelmingly positive. Seventy percent of the survey respondents indicated that they agreed that their expectations were met (47 of 67 ranking 4+ out of 5 with counts being 1, 7, 12, 29, and 18 respondents ranking 1 through 5, respectively). Eighty-two percent of the respondents indicated that our JupyterHub and the materials stored on it are useful tools for the learning process (54 of 66 marking agree or strongly agree with counts being 2 strongly disagree, 2 disagree, 8 neither agree nor disagree, 27 agree, and 27 strongly agree). Furthermore, the majority of our survey respondents indicated that their informatics expertise level increased after the boot camp concluded. This is exemplified by the fact that our respondents who considered themselves beginners decreased from 33.8 to 8.8% (see Fig. 1 ).

The results for the two questions concerning instructor responsiveness to questions and concerns can be found in Table 2 . Excluding N/A responses, 87.2% (41 of 47) indicated that our instructors had above average or better responsiveness during the lectures, and 86.1% (31 of 36) indicated that our instructors had above average or better responsiveness before and after lectures.

**Table 2:** Boot camp instructor responsiveness to participants' questions

Possible responses	Counts for instructor responsiveness during lectures	Counts for instructor responsiveness before and after lectures
Not responsive at all	0	1
Below average	0	0
Average	6	4
Above average	14	11
Extremely responsive	27	20
N/A (not applicable/relevant)	20	31

Forty-eight of the 68 respondents answered at least one of the free response questions. Analyses of these four questions showed several trends that were repeated throughout the respondents' answers. When asking about the expectations of the respondents, 4 stated the classes were too elementary, 9 stated that they were too difficult, often because they did not have enough background knowledge in biology and/or programming, 9 mentioned that they preferred an interactive experience and wished for more hands-on lectures in the program, 7 commented on the wide array of topics, 3 complained that the courses were not focused enough on clinicians/medical doctors, and 16 gave overall compliments or gratitude. We also noted that some individuals requested more one-on-one access to the professors. Finally, when the respondents were asked about subjects that they wanted to see covered in greater detail, four stated that they wanted additional classes on artificial intelligence/machine learning, five stated they wanted more on general coding (python, unix, R, etc.), and five stated that they wanted more classes on the implementations of biomedical informatic applications.

## Discussion

The boot camp was successful in its goal of exposing the participants to many different topics within the field of biomedical informatics. This is despite the challenges associated with teaching a wide array of courses to an audience of varying initial skill levels, as reflected by our conflicting results indicating difficulty levels that are both too low and too high. Further, the success of the JupyterHub was evident in the answers from the respondents. This is in-line with other studies that find JupyterHub to be a convenient teaching and learning tool for practical courses, especially those concerning coding. 15 16 The selected response analysis correlated with the free response analysis, as many respondents expressed a desire for more lectures or hands-on training on programming languages such as R and Python.

While we did see positive responses throughout the entirety of the program, we also noted a drop in the average number of participants per lecture ( Table 1 ). In fact, the first five lectures boasted the greatest number of live lecture participants. There are many factors that can affect these numbers, including the generalizability of the topic and the date and time of the course. Furthermore, participants may have been less inclined to join the live lectures once they realized that all lectures were recorded and available for asynchronous viewing.

Although 70% of the survey respondents indicated that they agreed that their expectations were met, there is still room for improvement. Of the 30% that did not indicate that their expectations were met, over half (12 of 20) stated that they did not agree or disagree. This correlates with the fact that many respondents indicated that they did not know what to expect when they first joined the program. While we attempted to give clear explanations of what we hoped to teach them when the boot camp began, this demonstrates the importance of clear communication of expectations and student learning objectives at the start of these programs, especially to diverse groups of learners.

The survey also allowed the respondents to self-evaluate their level of informatics comprehension before and after the boot camp ( Fig. 1 ). Possible answers were as follows: Beginner, Some Experience, Intermediate, Advanced, and Expert. We noted that the percentage of respondents that ranked themselves as beginners dropped from 33.8% to 8.8%. Each subsequent category increased in percentage, except for the expert category, indicating that the majority of the respondents stated that their informatics skills increased as a result of the boot camp. Giving participants the confidence to continue their informatics training was one of the goals of this boot camp.

We also allowed the respondents to rank our instructors' responsiveness to their questions and concerns both during and before/after the lectures ( Table 2 ). Excluding N/A responses, 87.2% (41 of 47) indicated that our instructors had above average or better responsiveness during the lectures, and 86.1% (31 of 36) indicated that our instructors had above average or better responsiveness before and after lectures. While these results are encouraging, it is important to note the large percentage of respondents who put N/A as their answers (∼30% for question 9 and 46% for question 10). This may indicate that the participants were not comfortable asking questions or that the difficulty level of the courses did not make them feel that questions were necessary. It may also indicate a lack of participant preparedness, which has already been indicated as a barrier to the integration of bioinformatics into life sciences education. 2

Limitations to our survey include that we did not collect demographic information from the participants as we were attempting to encourage as much participation as possible and wanted the respondents to feel that their answers were truly anonymous. However, without demographic data, we were unable to fully analyze the diversity of our participants. This prevents us from making adjustments that might support our diverse population by helping us adapt to different learning backgrounds. We will add additional questions concerning effective teaching styles for future QI surveys. We were also unable to analyze the retention level of topic-specific informatics skills. This would require multiple surveys with more specific questions about the content learned, potentially given after every course, especially given the wide array of topics offered.

## Conclusion

It was challenging to keep the difficulty level of lectures appropriate while utilizing multiple instructors with different teaching styles across numerous topics for an audience with varying initial skill levels. This correlates well with two of the grand challenges outlined by Işık et al., “supporting lifelong learning” and “training and equipping educators and trainers.” 1 Conflicting results were acquired from respondents as many individuals felt lectures were too difficult, while others felt they were too simple. Regardless, the survey results were taken into consideration, and additional lectures will be given in Python, R, and Unix programming in the future.

There are several other studies that investigate the efficacy of boot camp programs; overall, our results are in-line with the majority of these studies, reporting positive outcomes. 17 18 However, this boot camp was designed for a broad audience, whereas many other boot camp programs target a specific audience, such as only undergraduate students 19 or only postgraduate medical doctors. 20 This study also has the advantage of being highly flexible in that it is free and easily accessible both synchronously and asynchronously. The recordings are available long after the boot camp has concluded, an aspect that not all boot camps offer.

Finally, while the boot camp did have participants from all over the world with varying educational backgrounds, it is important to note that how one advertises and recruits for such educational opportunities can greatly affect the diversity of participants. 18

## Clinical Relevance Statement

The proper training of biomedical informaticians can and will save lives in clinical settings. Being able to appropriately analyze big data will allow future researchers to create novel treatment options and will lower the chance of harmful medical practices becoming mainstream due to misinformation. Teaching informatics comes with a litany of challenges that this study will help address.

## Multiple-Choice Questions

1. What was the main educational software environment used to store and implement materials for the University at Buffalo biomedical informatics boot camp?
  - Positron
  - Julia
  - JupyterHub
  - MatLab
  **Correct Answer** : The correct answer is option c. JupyterHub. This study utilized JupyterHub as a convenient educational environment for the participants. This JupyterHub contains a rich repository of information and codes designed to help teach biomedical informatics. While some of the codes stored here are used throughout the boot camp, there are additional codes, tutorials, data files, and lectures that serve as supplementary materials. These materials cover topics such as python and R coding, SPARQL Protocol and Resource Description Framework Query Language (SPARQL), general statistics including linear regression, natural language processing, and machine learning.
2. What was the majority response of the 68 survey respondents?
  - The boot camp was too elementary
  - Their skill level improved
  - They preferred practical lectures over theoretical lectures
  - Both b and c
  **Correct Answer** : The correct answer is option d. Both b and c are correct. Survey respondents indicated that their skill level improved through their answers to the selected response questions. They also indicated that they preferred practical lectures over theoretical lectures through their answers to the free response questions.
3. What was the topic that survey respondents wanted to be covered in more depth?
  - Ontology
  - Machine learning and AI
  - HIPAA standards
  - Genomic analyses
  **Correct Answer** : The correct answer is option b. Machine learning and AI. Survey respondents indicated that they wanted machine learning and AI to be covered in more depth through their answers to the free-response questions. They did not specifically indicate that they would like additional coverage on ontology, HIPAA standards, or genomic analyses.
