# Supplementary material for: Surveying the Efficacy of an Open Access Biomedical Informatics Boot Camp
Source: Appl Clin Inform. 2025 Jun 25;16(3):583–8. doi: 10.1055/a-2547-5208 (PMC12196558; doi:10.1055/a-2547-5208)
Supplement: Supplementary file 1 — Supplementary Material [file 10-1055-a-2547-5208-s202410soa0301.pdf]

Supplementary Table S1 Boot camp cross-sectional survey questions and corresponding responses

Question number	Question	Possible responses	Notes
Consent agreement	Do you consent to the answers you provide here being used in an academic study? Your participation in this survey is completely voluntary, and your responses are anonymous. You have the right to refuse to take part in this survey or choose to stop without judgment or penalty. You can choose to decline answering any particular question that you do not wish to answer for any reason.	1. Yes, I do<br>2. No, I do not	Answering “No, I do not” ends the survey
1	Please select the option that best describes your position.	1. Undergraduate<br>2. Master’s Graduate student<br>3. Doctoral level—PhD<br>4. Doctoral level—MD/DO<br>5. Doctoral level—MD/PhD<br>6. Doctoral level—DMD/DDS<br>7. Doctoral level—PharmD<br>8. Postdoctoral fellow<br>9. Clinical Informatics fellow<br>10. Faculty<br>11. Other:	“Other” option allows free response
2	Which of the following are you affiliated with? (Check all that apply.)	1. Academic Health Center<br>2. University<br>3. Community Hospital<br>4. Industry<br>5. Government<br>6. Other:	“Other” option allows free response
3	How many of the 24 lectures were you able to attend live or view the recording?	1. Only a few (1–4)<br>2. A little less than half (5–8)<br>3. Approximately half (9–13)<br>4. A little more than half (14–17)<br>5. Most of the lectures (18–23)	N/A
4a	How well did the BMI Boot Camp meet your expectations?	Linear scale of 1 (Much worse than expected) to 5 (Much better than expected)	N/A
4b	Please use the following space to elaborate on how your expectations were or were not met.	Free response	N/A
5	What subjects would you like to see covered that do not seem to be offered?	Free response	N/A
6	Please rate your level of informatics comprehension **before** the BMI Bootcamp.	1. Beginner<br>2. Some Experience<br>3. Intermediate<br>4. Advanced<br>5. Expert	N/A
7a	Please rate your level of informatics comprehension **after** the BMI Bootcamp.	1. Beginner<br>2. Some Experience<br>3. Intermediate<br>4. Advanced<br>5. Expert	N/A
7b	Do you agree that the BMI Bootcamp helped your understanding of specific informatics topics of special interest to you?	1. Strongly Disagree<br>2. Disagree<br>3. Neither Agree nor Disagree<br>4. Agree<br>5. Strongly Agree	N/A

(*Continued*)

 © 2025. The Author(s).

Supplementary Table S1 (*Continued*)

Question number	Question	Possible responses	Notes
8	Do you agree that our JupyterHub and the materials stored on it are useful tools for the learning process?	1. Strongly Disagree<br>2. Disagree<br>3. Neither Agree nor Disagree<br>4. Agree<br>5. Strongly Agree	N/A
9	How responsive were the instructors to your questions during lectures?	1. Not responsive at all<br>2. Below average<br>3. Average<br>4. Above average<br>5. Extremely responsive<br>6. N/A (not applicable or relevant)	N/A
10	How responsive were the instructors to your questions and concerns before and after lectures?	1. Not responsive at all<br>2. Below average<br>3. Average<br>4. Above average<br>5. Extremely responsive<br>6. N/A (not applicable or relevant)	N/A
11a	What would you change about the Boot camp to make its teaching informatics more effective?	Free response	N/A
11b	What would you change about the Boot camp to make its teaching of coding more effective?	Free response	N/A

 © 2025. The Author(s).
